# Transcriptome sequencing of two parental lines of cabbage (*Brassica oleracea* L. var. *capitata* L.) and construction of an EST-based genetic map

**DOI:** 10.1186/1471-2164-15-149

**Published:** 2014-02-22

**Authors:** Nur Kholilatul Izzah, Jonghoon Lee, Murukarthick Jayakodi, Sampath Perumal, Mina Jin, Beom-Seok Park, Kyounggu Ahn, Tae-Jin Yang

**Affiliations:** 1 Department of Plant Science, Plant Genomics and Breeding Institute, and Research Institute for Agriculture and Life Sciences, College of Agriculture and Life Sciences, Seoul National University, Seoul 151-921, Republic of Korea; 2 National Institute of Agricultural Biotechnology, Rural development Administration, Suwon 441-707, Korea; 3 Joeun Seed, #174, Munbang-Ri, Cheonhan-Myun, Goesan-Gu, Chungcheongbuk-Do 367-833, Korea; 4 Present Address: Indonesian Research Institute for Industrial and Beverage Crops (IRIIBC), Pakuwon, Sukabumi, Indonesia

**Keywords:** Cabbage, EST, Genetic linkage map, SSR, SNP, Transcriptome sequencing

## Abstract

**Background:**

Expressed sequence tag (EST)-based markers are preferred because they reflect transcribed portions of the genome. We report the development of simple sequence repeat (SSR) and single nucleotide polymorphism (SNP) markers derived from transcriptome sequences in cabbage, and their utility for map construction.

**Results:**

Transcriptome sequences were obtained from two cabbage parental lines, C1184 and C1234, which are susceptible and resistant to black rot disease, respectively, using the 454 platform. A total of 92,255 and 127,522 reads were generated and clustered into 34,688 and 40,947 unigenes, respectively. We identified 2,405 SSR motifs from the unigenes of the black rot-resistant parent C1234. Trinucleotide motifs were the most abundant (66.15%) among the repeat motifs. In addition, 1,167 SNPs were detected between the two parental lines. A total of 937 EST-based SSR and 97 SNP-based dCAPS markers were designed and used for detection of polymorphism between parents. Using an F_2_ population, we built a genetic map comprising 265 loci, and consisting of 98 EST-based SSRs, 21 SNP-based dCAPS, 55 IBP markers derived from *B. rapa* genome sequence and 91 public SSRs, distributed on nine linkage groups spanning a total of 1,331.88 cM with an average distance of 5.03 cM between adjacent loci. The parental lines used in this study are elite breeding lines with little genetic diversity; therefore, the markers that mapped in our genetic map will have broad spectrum utility.

**Conclusions:**

This genetic map provides additional genetic information to the existing *B. oleracea* map. Moreover, the new set of EST-based SSR and dCAPS markers developed herein is a valuable resource for genetic studies and will facilitate cabbage breeding. Additionally, this study demonstrates the usefulness of NGS transcriptomes for the development of genetic maps even with little genetic diversity in the mapping population.

## Background

The genus *Brassica* includes some of the most economically important crops with wide-ranging adaptability for cultivation under various agro-climatic conditions. Among these are six widely cultivated species, including three monogenomic diploids: *B. rapa* (AA, 2*n* = 20), *B. nigra* (BB, 2*n* = 16), *B. oleracea* (CC, 2*n* = 18); and three amphidiploids (allotetraploids): *B. juncea* (AABB, 2*n* = 36), *B. napus* (AACC, 2*n* = 38) and *B. carinata* (BBCC, 2 *n* = 34), which evolved through hybridization between different monogenomic diploids as described by U’s triangle [1]. Among those cultivated species, *B. oleracea* exhibits the largest genetic and morphological diversity [2], encompassing many common vegetables such as cabbage, broccoli, cauliflower, Brussels sprout, kale, kohlrabi, and kai-lan.

In recent years, development of massive sequencing technology has led to new possibilities for high-throughput genome analysis [3]. Transcriptome sequencing has become an attractive approach compared to whole-genome sequencing because it allows efforts to be targeted to genic regions [4]. The advent of transcriptome sequencing has yielded a huge amount of transcribed sequence data, such as expressed sequence tags (ESTs) that can be exploited for gene expression profiling, genome annotation, comparative genomics and physical mapping. ESTs offer a simple strategy to study the transcribed portions of genomes and provide a robust sequence resource from which to develop functional markers [5,6].

Among next-generation sequencing (NGS) technologies, the 454 sequencing platform has been successfully employed for *de novo* transcriptome sequencing of many plant species, including American ginseng (*Panax quinquefolius*) [7], *A. thaliana*[8], maize (*Zea mays*) [9] and olive (*Olea europaea*) [10]. A single-plate run on the 454 GS-FLX titanium platform typically produces around million reads with an average length of 400 bp, and is faster and cheaper than traditional Sanger sequencing methods [11].

Molecular DNA markers have greatly contributed to the development of plant genetics and breeding studies. The use of DNA markers has become essential for crop improvement programs, such as for cultivar identification, genetic diversity, linkage map construction and identification of quantitative trait loci (QTL) [12]. Among the many types of DNA markers, simple sequence repeats (SSRs) and single nucleotide polymorphisms (SNPs) are the preferred marker types for many genetic applications. SSRs are efficient co-dominant anchor markers with high levels of polymorphism and can easily be amplified by polymerase chain reaction (PCR) using primers designed from flanking sequences of the SSR motifs. Meanwhile, SNPs are abundant in virtually all populations, with the majority being biallelic, and can be tightly linked to or are the actual cause of allelic (phenotypic) differences in traits [13].

A total of 19 different genetic linkage maps of *B. oleracea* have been published so far [14]. However, most of those maps were developed using RFLP markers, which could not be easily transferred to other genetic maps. In addition, only a few functional markers have been mapped in *B. oleracea*. The latest cabbage genetic map constructed by Wang et al. [14] contained 80 EST-SSR markers. However, publicly available EST-based SSR and EST-based dCAPS markers are still limited in this plant species. Hence, development of more functional markers is desirable in order to facilitate the mapping, tagging and identification of important trait loci.

To generate large-scale EST data and to develop functional markers using ESTs, we performed large-scale transcriptome sequencing of two cabbage parental lines, C1184 and C1234, which have been used as elite breeding lines for development of commercial F_1_ varieties by the Joeun Seed company in Korea, using the 454 sequencing platform, Roche GS FLX Titanium series. These newly developed EST-based SSR and dCAPS markers were mapped along with SSR markers and intron-based polymorphism (IBP) markers that were previously reported for *Brassica* species. This genetic map will promote QTL mapping and breeding.

## Results

### Sequence assembly and functional annotation

**Figure 1:**
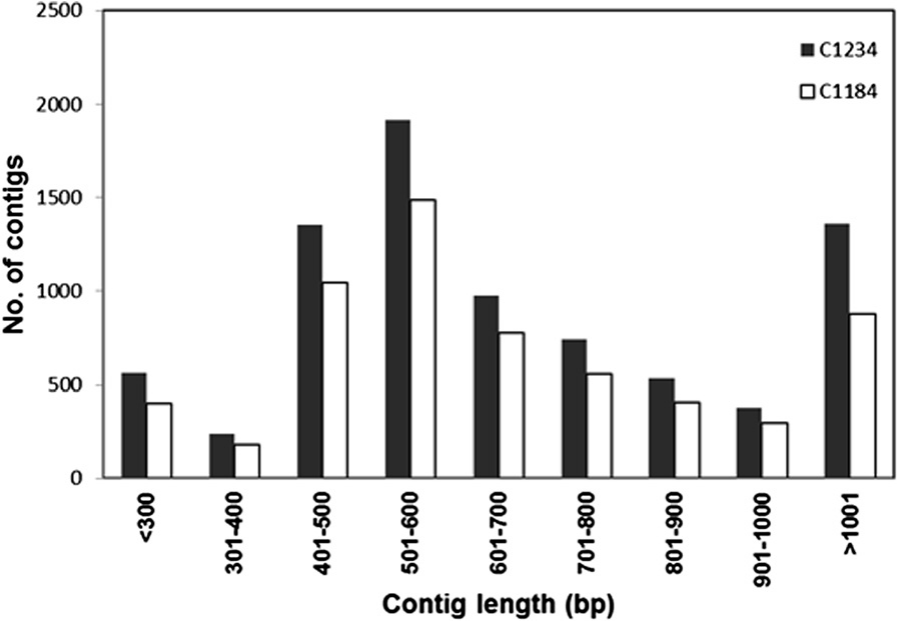
Size distribution of the contigs.

We obtained 92,255 and 127,522 high quality (HQ) reads for the C1184 and C1234 cabbage parental lines, respectively. *De novo* assemblies generated 34,688 unigenes including 6,037 contigs and 28,651 singlets for C1184, and 40,947 unigenes that contained 8,068 contigs and 32,879 singlets for C1234. In total, 63,604 and 94,643 reads of C1184 and C1234, respectively, were assembled into contigs, accounting for 68.94% and 74.22% of all sequencing reads. The majority of these contigs were in the range of 501–600 bp, with an average size of 693 bp and 730 bp for the C1184 and C1234 lines, respectively (Figure 1; Table 1).

**Table 1:** Summary of 454 transcriptome sequencing and assembly data

**Parameters**	**C1184**	**C1234**
Response to black rot disease	Susceptible	Resistant
No. of HQ reads	92,255	127,522
Total number of assembled contigs	6,037	8,068
No. of singletons	28,651	32,879
No. of unigenes	34,688	40,947
Largest contig length (bp)	3,820	6,337
Average contig length (bp)	693	730
N50 length (bp)	734	781

Prior to functional annotation, the singletons from both lines that were less than 200 bp in length were removed. The remaining 33,244 and 38,088 assembled unique transcripts found in cabbage lines C1184 and C1234, respectively, were compared against the NCBI non-redundant (nr) protein database using the BLASTX algorithm. Of these, 27,740 (79%) of the C1184 unigenes and 31,458 (76%) of the C1234 unigenes had significant hits. Among the transcripts with hits, more than 76% matched known functional genes in both lines.

**Figure 2:**
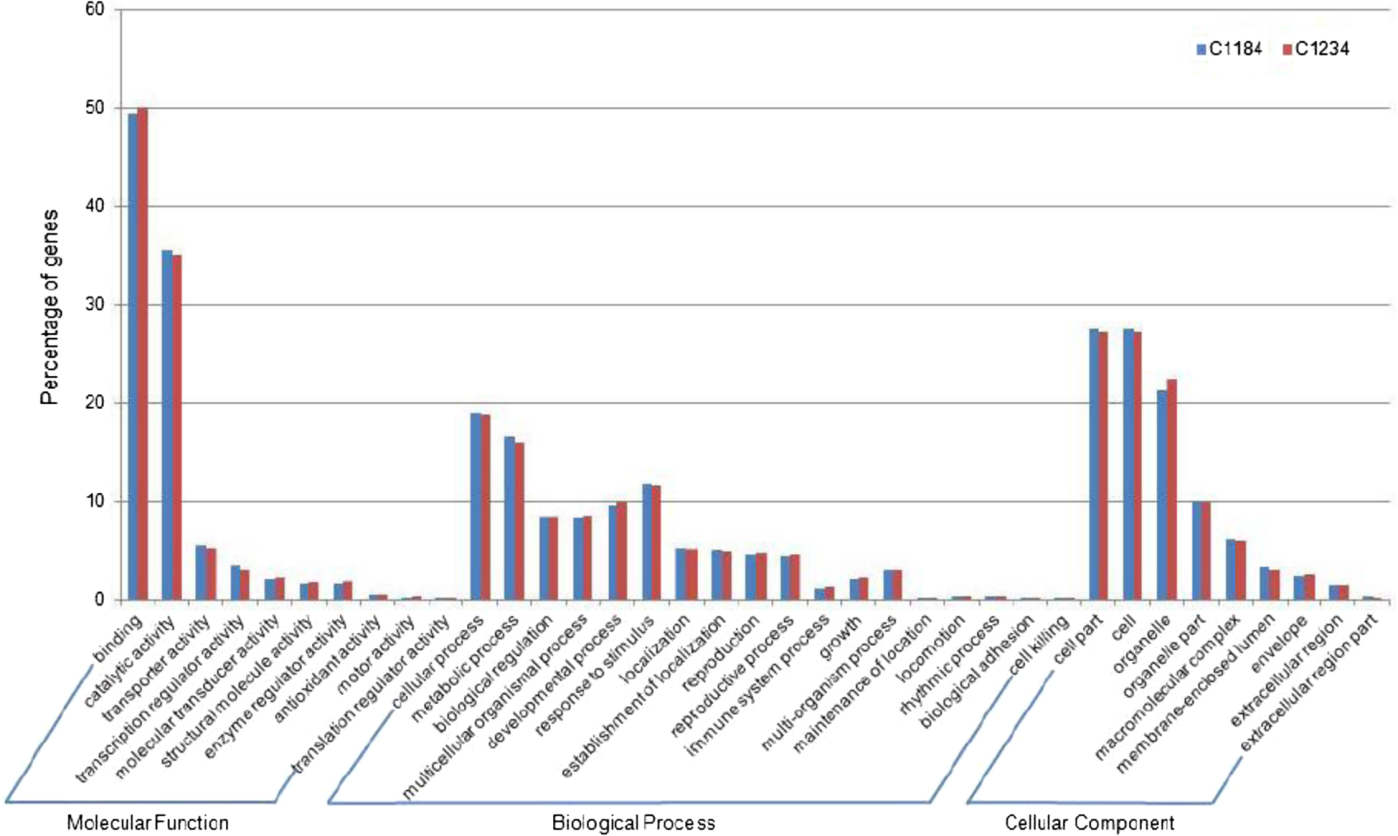
**Functional classifications of C1184 and C1234 unigenes.** Percentage of cabbage unigenes classified into different functional categories of level 2 GO.

To explore and summarize the functional categories of the unigenes, we used Blast2GO to obtain the Gene Ontology (GO) terms for the representation of molecular function, cellular component and biological process. Approximately 24,931 (90%) and 28,093 (89%) of C1184 and C1234 unigenes that had BLAST hits, respectively, could be assigned to one or more ontologies. The results corresponded to a wide diversity of functional categories in all levels of the Gene Ontology database. Figure 2 shows the unigene distribution for three main categories under GO level 2.

### NBS-encoding genes in the black rot-resistant line

Most disease resistance R genes encode putative proteins containing nucleotide binding sites (NBS) and leucine-rich repeat (LRR) domains. NBS-LRR genes can be divided into the Toll-interleukin-1 receptor (TIR-NBS-LRR) and coiled-coil (CC-NBS-LRR) sub-families. Plant NBS-LRR-encoding genes play an important role in the responses of plants to various pathogens, including viruses, bacteria and fungi. Hence, the black rot-resistant line C1234 was searched for NBS-encoding genes. A total of 29 ESTs related to the NBS-LRR domain were identified in the C1234 line (Additional file 1). Among them, 22 were classified as TIR-NBS-LRRs and 7 were categorized as CC-NBS-LLRs. Thus, we conclude that many of the NBS genes in *B. oleracea* belong to the TIR-NBS-LRR type. Further, we compared the abundance/expression profile of NBS genes between C1234 and C1184 lines based on the mapping read count. As expected, we found a higher number of mapping counts for NBS genes in the resistant line (C1234) when compared to the susceptible (C1184) line (Additional file 1).

### Frequency and distribution of SSRs in cabbage ESTs

In the 40,947 unigene set of C1234, a total of 2,405 SSRs were identified from 2,214 unique ESTs with an average of one SSR per 20 ESTs. 96 ESTs were found to have more than two SSRs and 95 contained compound formations (Table 2). The compound formations comprised either more than one repeat motif or the same repeat motif interrupted by a short non-repetitive sequence.

**Table 2:** Summary of EST-based SSRs identified

**Investigated elements**	**Number**
Total EST sequences examined	40,947
Total SSRs	2,405
- Dinucleotide motif	750
- Trinucleotide motif	1,591
- Tetranucleotide motif	24
- Pentanucleotide motif	12
- Hexanucleotide motif	28
ESTs containing SSRs (SSR-ESTs)	2,214
- ESTs containing 1 SSR	2,023
- ESTs containing more than 2 SSRs	96
- ESTs with SSRs in compound formation	95
SSR primer design	
- ESTs used for SSR primer design	937
- ESTs excluded for primer design	
· exact match between both parents	740
· short flanking sequence insufficient to design primers	624

Trinucleotide repeats were the most abundant type (1,591, 66.15%), followed by dinucleotide repeats (750, 31.19%). The other repeat types, including tetra-, penta- and hexa-nucleotide represented less than 2% of the SSRs identified (Table 2). Among trinucleotide repeats, the most plentiful was AAG/AGA/GAA (484, 20.12%), followed by the ATC/TCA/CAT (299, 12.43%) and AGG/GGA/GAG (228, 9.48%). The AG/GA motif (543, 22.58%) was the most common among the dinucleotide repeats, while AT/TA (134, 5.57%) and AC/CA (73, 3.04%) motifs were much less frequent (Table 3).

**Table 3:** Characteristics of EST-SSRs and efficiency of marker development in cabbage

**Motif**	**No. of EST-SSRs (%)**	**No. of designed primer pairs**	**No. of primer pairs amplifying product (%)**^**a**^	**No. of polymorphic primers (%)**^**b**^
Dinucleotide	750 (31.19)	223	183 (82.06)	26 (14.21)
AC/CA	73 (3.04)	29	26 (89.66)	4 (15.38)
AG/GA	543 (22.58)	162	130 (80.25)	18 (13.85)
AT/TA	134 (5.57)	32	27 (84.38)	4 (14.81)
Trinucleotide	1,591 (66.15)	645	589 (91.32)	78 (13.24)
AAC/ACA/CAA	151 (6.28)	64	57 (89.06)	9 (15.79)
AAG/AGA/GAA	484 (20.12)	195	171 (87.69)	20 (11.70)
AAT/ATA/TAA	26 (1.08)	11	10 (90.91)	2 (20)
ACC/CAC/CCA	143 (5.95)	60	57 (95)	10 (17.54)
ACG/CGA/GAC	25 (1.04)	7	7 (100)	-
ACT/CTA/TAC	39 (1.62)	19	16 (84.21)	2 (12.5)
AGC/GCA/CAG	135 (5.61)	52	50 (96.15)	5 (10)
AGG/GGA/GAG	228 (9.48)	103	98 (95.15)	15 (15.31)
ATC/TCA/CAT	299 (12.43)	111	101 (90.99)	13 (12.87)
CCG/CGC/GCC	61 (2.54)	23	22 (95.65)	2 (9.1)
Tetranucleotide	24 (1)	15	13 (86.67)	2 (15.38)
Pentanucleotide	12 (0.5)	4	3 (75)	1 (33.33)
Hexanucleotide	28 (1.16)	11	10 (90.91)	2 (20)
Compound formation	92	39	31 (79.49)	7 (22.58)
Total	2405 (100)	937	829 (88.47)	116 (13.99)

^a^Percentage of designed primer pairs successfully amplifying EST-SSRs.

^b^Of the primer pairs that amplified product, percentage showing polymorphism.

### Development of EST-based SSR markers

From the 2,214 ESTs containing SSRs, we designed a total of 937 EST-based SSR markers. The remaining 1,677 ESTs were excluded for SSR marker development due to short flanking sequence insufficient for primer design (624 ESTs) or because they had identical sequences in both parents (740 ESTs). The 937 EST-based SSR markers were used for a parental polymorphism survey between lines C1184 and C1234. Successful amplification was obtained from 829 primer sets (88.47%). Polymorphism was identified from 116 (13.99%) primer sets (Additional file 2), and we used 99 SSR markers for further mapping after excluding those giving rise to unclear band patterns and dominant marker types (Table 3). The majority of the primer pairs amplified a single polymorphic locus, except for BoESSR045, which had two polymorphic loci.

**Figure 3:**
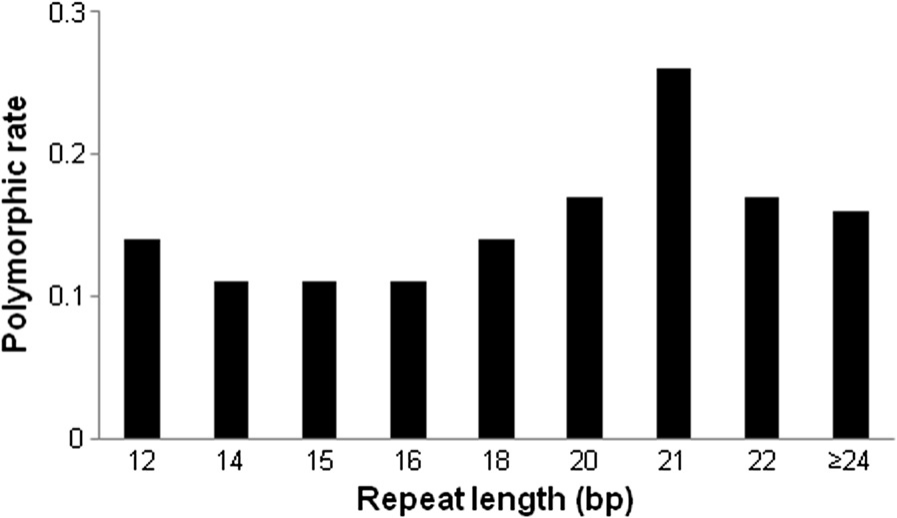
**Relationship between repeat length and polymorphic rate.** Polymorphic rate was calculated as polymorphic markers per primer pair that successfully amplified product.

The rate of successful amplification and polymorphism did not significantly vary with SSR motif length (Table 3). On the other hand, the primers for SSRs of more than 20 bp showed a much rate of higher polymorphism (17.03%) than those for SSRs of less than 18 bp (11.59%) (Figure 3).

### Development of EST-based dCAPS markers

Potential SNPs were detected by mapping C1234 raw reads onto C1184 contigs by CLC mapping. A total of 1,167 SNPs including 703 transitions and 464 transversions as well as 160 INDELs were identified (Table 4). Regarding transition type SNPs, the A/G type (341, 48.51%) was found to have slightly lower frequency than the C/T type (362, 51.49%). Meanwhile, for transversion type SNPs, A/T (128, 27.59%) was the most common and A/C (106, 22.84%) was the least common. Of the candidate SNPs, 97 were used for development of dCAPS markers. Among them, 90 markers successfully amplified product and 49 markers (54.44%) showed polymorphism between the two parental lines (Additional file 3). We subsequently used 21 of these dCAPS markers for genotyping F_2_ populations.

**Table 4:** Summary of SNPs between homologous EST pairs from two cabbage lines

**Type of SNP**		**Number**
Transition	A < − > G	341
	C < − > T	362
	Total	703
Transversion	A < − > T	128
	G < − > T	110
	G < − > C	120
	A < − > C	106
	Total	464
Total SNPs		1,167
INDELs		160
Total		1,327

### Construction of genetic linkage map

**Figure 4:**
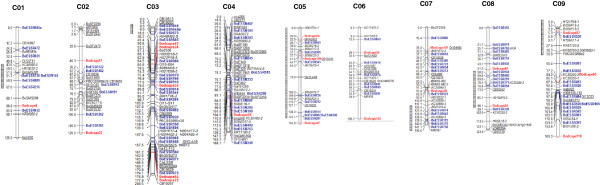
**The genetic linkage map of cabbage.** The map was constructed using 98 new EST-based SSR marker loci named “BoESSR” (blue), 21 new EST-based dCAPS markers named “BodCAPS” (red), 91 published SSR markers (anchor markers are underlined), and 55 reported Br-IBP markers (italics). The bar on left of the genetic linkage map indicates segregation distortion regions (SDRs).

A total of 120 polymorphic marker loci, including 99 EST-based SSR and 21 SNP-based dCAPS marker loci were used along with 151 previously reported markers to construct a genetic map. The linkage map represents 265 loci assigned to 9 linkage groups (LGs), in accord with the haploid number of cabbage chromosomes (2*n* = 18, *n* = 9), and designated as C01-C09 (Figure 4). Only seven markers (2.57%) failed to be placed on the map. The linkage map created here covered 1,331.88 cM with an average distance between neighboring loci of 5.03 cM. Among the mapped loci, 75 have previously been placed on *Brassica* genetic maps [14-21], and were used as anchoring markers for the reference map. However, we found that six anchor markers were mapped into different LGs in this study. Markers BnGMS299, BoE506, BoSF2369, Ol10-B01 were previously mapped to LG 9, 4, 2 and 7, respectively [14,16,20], but mapped to C01, C03, C07 and C04, respectively, in this study. Likewise, markers sA34 and CB10267 were mapped to LG 1 previously [18,19] but positioned on C08 and C03, respectively, in this current study. The newly developed EST-based markers were distributed across all nine LGs. LG C03 had the most mapped EST-based SSR loci (20), whereas C02 and C06 had the least (6). Meanwhile, the number of mapped loci for EST-based dCAPS markers ranged from 1 in C01 and C04 to 5 in C03. Overall, C03 was also the largest LG, including 52 loci and spanning 208.515 cM. C01 contained the fewest mapped loci (18), although its map length (125.00 cM) was longer than that of C06 (106.32 cM), which comprised 19 mapped loci. The average distance between adjacent markers ranged from 3.93 (C04) to 6.94 (C01) (Table 5).

**Table 5:** Distribution of molecular markers on the cabbage genetic map

**Linkage group**	**Length (cM)**	**No. of mapped loci**						**Average distance between adjacent loci (cM)**	**Number of gaps (> 20 cM)**
		Previously reported*			**This study**				
		**EST-SSR**	**gSSR**	**Br-IBP**	**EST-SSR**	**EST-dCAPS**	**Total**		
C01	124.996	0	6	3	8	1	18	6.94	1
C02	129.508	0	16	1	6	2	25	5.18	0
C03	208.515	1	16	10	20	5	52	4.01	1
C04	169.057	4	18	7	13	1	43	3.93	1
C05	154.898	0	4	7	9	3	23	6.73	3
C06	106.32	0	8	3	6	2	19	5.59	1
C07	145.632	0	8	7	12	2	29	5.02	1
C08	123.619	0	5	6	12	2	25	4.94	1
C09	169.33	0	5	11	12	3	31	5.46	3
Total	1,331.88	5	86	55	98	21	265	5.03	12

*Previously reported markers are from Wang et al. [14], Mun et al. [22], Lowe et al. [16], Piquemal et al. [17], Radoev et al. [19], Cheng et al. [20], Uzunova and Ecke [23], Burgess et al. [24], Suwabe et al. [25], Kim et al. [21], Long et al. [18], Louarn et al. [26] and Smith and King [15].

We identified some large gaps throughout the LGs. Twelve gaps with >20 cM between adjacent markers were identified in eight LGs (Table 5). C05 and C09 were each found to have three gaps in their LGs. The largest gaps were detected in C03, with 30.6 cM between BodCAPS22 and CB10267. This indicates that the marker loci were unevenly distributed in the nine LGs of the cabbage genetic map.

### Segregation distortion of polymorphic markers

Segregation distortion is defined as the phenomenon that alleles at a locus deviate from the Mendelian expectation [27]. The occurrences of segregation distortion have been observed in *Brassica* species which showed a number of distorted markers mapped on the genetic map [14,20,28]. In this study, we assigned all but 7 of the 271 polymorphic markers to linkage groups. Most of the mapped markers segregated with the expected 1:2:1 Mendelian ratio in the F_2_ population. However, 68 (25.66%) markers showed a segregation pattern distorted from this ratio (Table 6). These distorted markers were clustered or scattered in all LGs except in C06. The clusters of more than three distorted markers were designated segregation distortion regions (SDRs). Of the nine LGs, we were able to detect SDRs in six. The longest SDR was found in C05, with 20 markers spanning about 143.08 cM and covering 86.96% of C05. Meanwhile, the shortest SDR spanned 9.47 cM in C03, with only 3 markers identified (Table 7).

**Table 6:** Features of the molecular markers used for mapping

**Marker type**		**No. of primers used for polymorphic survey**	**No. of polymorphic primers**	**No. of mapped markers**	**No. of unlinked markers**	**No. of distorted markers**
Previously reported*	gSSR	657	88	86	2	11
	EST-SSR	38	5	5	0	0
	Br-IBP	1,841	58	55	3	22
This study	EST-SSR	937	99	97	2	27
	EST-dCAPS	97	21	21	0	8
Total		3,570	271	264	7	68

*Previously reported markers were used from Wang et al. [14], Mun et al. [22], Lowe et al. [16], Piquemal et al. [17], Cheng et al. [20], Uzunova and Ecke [23], Varghese et al. [29], Cheng et al. [20], Burgess et al. [24], Suwabe et al. [25], Kim et al. [21], Choi et al. [30], Batley et al. [31], Long et al. [18], Iniguez-Luy et al. [32], Kresovich et al. [33], Szewc-McFadden et al. [34], Louarn et al. [26], Bell and Ecker [35], Lagercrantz et al. [36], Sebastian et al. [37], Smith and King [15], HRI (unpublished) and Saito et al. [38].

**Table 7:** Distribution of molecular markers in the segregation distortion regions of the cabbage linkage map

**Linkage group**	**No. of distorted markers**	**No. of distorted markers located in SDRs**^**a**^	**Length of SDRs (cM)**
C01	9	7	23.97
C02	1	0	0
C03	6	3	9.47
C04	3	0	0
C05	20	20	143.08
C06	0	0	0
C07	9	8	19.21
C08	10	9	37.85
C09	10	10	38.94
Total	68	57	272.52

^a^*SDRs*, segregation distortion regions.

## Discussion

### Transcriptome sequencing, assembly and gene annotation

Transcriptome sequencing has proven to be an important tool for gene discovery, allele mining and marker development. In this study, the 454 GS-FLX platform was utilized due to its longer read length, which enables high-quality *de novo* assembly of the transcriptome without a characterized reference genome [39]. Additionally, Newbler v.2.3 software, which is currently the most robust software for 454 transcriptome assembly [40], was chosen for assembling the sequence reads. Consequently, a vast quantity of potential unique ESTs were generated, representing a large fraction of the cabbage transcriptome, and were further used for development of SSR and SNP markers. The quality of the sequence information obtained here was confirmed by the high percentage of unigenes matching to known proteins by BLASTX and the high rate of successful PCR amplifications.

In order to examine the potential functions represented in the cabbage transcriptome, BLASTX analyses were performed against NCBI non-redundant protein database. At first, we removed singletons shorter than 200 bp because the percentage of BLAST hits usually shows positive correlation with sequence length, as has been confirmed previously in sweet potato [41] and celery [42]. The BLASTX results revealed that more than 76% of the cabbage transcripts had similarity to known unique proteins. In addition, a large proportion of transcripts (± 89%) were assigned to a remarkable range of GO categories (Figure 2), indicative of the diversity of genes represented by the cabbage transcriptome. Of all assignments made in three categories, most mapped into the GO terms of binding activity (49.5%) and catalytic activity (35.1%) under the molecular function category. Our results for GO term distribution are in agreement with previous studies in rubber tree and pea transcriptome sequencing [43,44]. Further, we identified and analyzed NBS-encoding disease resistance genes. Although 454 technologies have low sequencing depth, we estimated the NBS-LRR gene expression profiles based on read count. In general, the resistant line showed higher expression of NBS-LRR genes than did the susceptible line. These findings contribute to understanding the evolution of NBS-encoding genes in *Brassica* species.

### General features of EST-SSRs in the cabbage genome

The large collection of EST sequences generated in the present study facilitates the identification of SSRs by *in silico* mining, which requires relatively little time and has been applied in a variety of plant species [45]. Approximately 5.41% of the 40,947 cabbage unigenes possessed at least one SSR, which is in accord with values reported for other species, ranging from ~2 to ~16% [46-48]. The EST-SSR frequency observed here was higher than previous reports for *A. thaliana*, maize, tomato, cotton, poplar, and flax [49,50]. However, it is important to note that values for SSR abundance and frequency among different plant ESTs significantly depend on the parameters used to detect SSRs, the size of the dataset, the database mining tools, and the EST sequence redundancy [45,51].

Our investigation revealed that trinucleotide repeats are the most common repeat motif attributed to the fact that they can generate non-frame-shift mutations in the coding region [52], and perhaps result in variation of amino acid residue number at the protein level [50]. Earlier studies demonstrated that AG/CT and AAG/CTT were the predominant di- and trinucleotide SSR motifs, respectively, in plant dicot ESTs [2,14,50,51,53,54], which is similar to our observation. These findings suggest that AG and AAG motifs can be considered common features of EST-SSRs in dicot plants.

### Marker development and polymorphism level of EST-based SSR markers

EST-SSRs are known to have high level of transferability across taxa and could be useful as anchor markers for comparative mapping and evolutionary studies [45]. In present study, a new set of 937 EST-based SSR markers was developed and 88.47% of them yielded amplification products. The amplification rate observed here is slightly higher than that reported for sweet potato (84.6%) [51] and tomato (83%) [55], but lower than that for *B. rapa* (97.74%) [2]. The success rate for SSR amplification generally ranges between 60-90%, as previously reported for several crop plants [45].

The EST-based markers designed here showed low polymorphism, which reflects the fact that EST-SSR markers have less polymorphism than genomic SSRs due to highly conserved DNA sequences in genic regions [45,56]. Another factor likely contributing to the low level of polymorphism is that the parental lines used in this study are elite breeding lines that have a close genetic relationship. Even though EST-based SSR markers exhibit relatively low polymorphism, they may be linked to candidate genes or a trait of interest [57], and as such can be more valuable than anonymous markers. Moreover, markers designed from compound formations exhibited the highest level of polymorphism. This could be due to compound formations containing more than one SSR motif, which could increase the probability of polymorphism. In addition, previous studies reported that the level of polymorphism of SSR markers is usually correlated with SSR length, as observed in pepper and rice [53,58]. Likewise, in this study, higher polymorphism was also observed when EST-SSR markers included more than 20 bp of SSR length.

### Validation and polymorphism analysis of EST-based dCAPS markers

A considerable number of SNPs were successfully identified and used to design dCAPS markers. The dCAPS method creates polymorphism from SNPs by restriction endonuclease digestion of the PCR products [59]. Approximately 54.44% of the amplified primers showed polymorphism between the two parental lines. The remaining 46% did not show any polymorphism that might be derived from sequencing errors or mis-alignment between paralogous genes of the triplicated *Brassica* genome [60,61]. The polymorphism analysis obtained in this study was in accordance with a previous study in cabbage by Wang et al. [14], in which it was also observed that SNP markers had higher polymorphism than SSR markers. The combination of SSR and SNP markers designed here allowed a larger number of EST-based markers to be mapped onto the cabbage genetic linkage map.

### Linkage map construction for cabbage

We effectively constructed a genetic linkage map for cabbage spanning a total 1,331.88 cM, which is slightly larger than the earlier cabbage genetic map (1197.9 cM) generated by Wang et al. [14]. The difference in length between these two maps could be contributed by the difference in the chromosomal recombination frequency caused by environmental factors and the genetic distance between mapping parents, and also the different size of the mapping population as well as the number and types of markers used for map construction [54].

Some of the mapped EST-based marker loci were found to be clustered in narrow regions, e.g. C03 (6 loci within 6.3 cM), CO7 (3 loci within 2.2 cM) and C08 (3 loci within 1.1 cM). This clustering might correspond to the gene-rich regions of cabbage. Clustered markers in genetic maps were also reported in soybean [62], pepper [53], and *B. rapa*[2], as well as a previous cabbage map [14]. In addition, we observed 12 gaps in total along this map that varied in size (>20 cM). These gaps were detected in all LGs except LG 2, suggesting that such gaps are not restricted to a particular region of the chromosomes. The presence of these gaps may have negative effects on the application of mapped DNA markers. As mentioned by Cregan et al. [63], genomic regions that lack DNA markers will make detection of quantitative trait loci (QTL) difficult. Therefore, we plan to develop more markers in the near future to fill in gaps between markers and achieve a high-density genetic linkage map. We also observed that six markers that we used as anchor markers were mapped to the different LGs than previously reported. This could be due to some of those markers producing multiple bands, which could lead to a band derived from a paralogous locus being mapped in our population. Another possible explanation is due to genomic rearrangement which may be observed in genetically unstable population such as in F_2_ population that we used in this study. As also reported by Wang et al. [14] that F_2_ population are temporary and difficult to maintain for long term period.

### Segregation distortion phenomenon in the cabbage genetic map

Segregation distortion is a common fact in segregated populations generated from crosses between diverse genotypes [64,65]. In plants, segregation distortion was first reported in maize [66], and subsequently in many species including rice [67], wheat [68], Arabidopsis [69], and cabbage [14]. Segregation distortion can have important implications for the construction of a genetic map and QTL mapping, but if addressed properly, distorted markers can also be helpful for QTL mapping [70]. Many factors such as mapping population type, marker type, and genetic relationships of the parents are closely related to the extent of segregation distortion [71].

A recent study identified 26 SDRs on seven LGs of a cabbage genetic map [14]. In the present study, we detected six SDRs located on six LGs, specifically C01, C03, C05, C07, C08, and C09. The difference in SDR numbers may be related to the different types of mapping population used for constructing the maps, since for the earlier map they used a double haploid (DH) population, whereas in this study an F_2_ population between elite breeding lines was used. Thus, this finding is in strong agreement with those of Zhang et al. [72] that segregation distortion is more frequent in DHs and RILs than F_2_ populations. Moreover, we found that the longest distorted regions were on C05. This result reflects that the distorted markers were non-randomly distributed throughout the genome [73]. Additionally, the fact that markers with segregation distortion are clustered in particular regions indicates that segregation distortion in the F_2_ population is most likely caused by genetic factors and unlikely to be due to statistical bias, genotyping or scoring errors [74]. The existence of SDRs suggests that there has been a selective process in gametophytes or sporophytes [75]. Also, based on studies in other crops, SDR loci may be linked to sterility genes and pollen-suppressed genes that can affect the selection of partial gametophytes or sporophytes [14]. Overall, the results represent an initial finding of segregation distortion in cabbage; therefore, further investigation is needed to understand better the mechanism underlying the segregation distortion phenomenon in the cabbage genetic map.

## Conclusions

The 454 GS-FLX platform has been established to be a powerful tool for *de novo* transcriptome sequencing due to its long read length. A large number of cabbage EST sequences were generated and used as a reliable source for marker development and discovery of a new candidate disease resistance gene. Subsequently, a novel set of 937 SSR and 97 dCAPS markers were successfully developed and validated using two parental lines of cabbage. Of these, 99 SSR and 21 dCAPS markers revealed clear polymorphism between the two cabbage parental lines, and together with previously developed markers were used to construct a genetic linkage map for cabbage. The map generated herein will facilitate the identification of candidate QTL for economically important traits. In addition, these newly developed markers increase the publicly available EST-based markers in cabbage, which readily can be utilized for other *Brassica* species. This result demonstrates that transcriptome sequencing using the 454 GS-FLX Titanium sequencer can be a fast and efficient approach for gene discovery and marker development, especially for species without reference genome sequence.

## Methods

### Plant materials and genomic DNA extraction

Young leaf samples of two cabbages parental lines, C1184 and C1234, were collected for RNA extraction. The samples were immediately frozen in liquid nitrogen and stored at −70°C until use. For construction of a genetic linkage map, 97 F_2_ plants were developed from a cross between C1184 as the female parent and C1234 as the male parent. These two cabbage inbred lines were selected because they are relatively diverse among 16 inbred lines bred for F_1_ cultivar development in the Joeun Seed company in Korea after a study on their genetic distance based on SSR markers used in a previous report [76]. Additionally, they show different responses to black rot disease: C1184 is susceptible, while C1234 is resistant. All plant materials used in this study were kindly provided by Joeun Seeds, Chungcheongbuk-Do, Korea.

The total genomic DNA was extracted from the leaves of each F_2_ plant according to the modified cetyltrimethylammonium bromide (CTAB) method [77]. The quality and quantity of the extracted DNA were estimated with a NanoDrop ND-1000 (NanoDrop Technologies, Inc., Wilmington, DE, USA). The final concentration of each DNA sample was adjusted to 10 ng/μL for PCR analysis.

### 454 transcriptome sequencing and assembly

Total RNA was extracted from approximately 5 g leaf tissue of cabbage C1184 and C1234 using the SV Total RNA Isolation Kit (Promega, Madison, WI) according to the manufacturer’s instructions. cDNA synthesis and library construction from 5 μg extracted mRNAs was then performed as described in the cDNA Rapid Library Preparation Method Manual provided with the Roche GS FLX Titanium Series. Total RNAs were fragmented using a 96 ring Magnetic Particle Concentrator (MPC), and double-stranded cDNA was then synthesized with the cDNA Synthesis System Kit (Roche, IN, USA). Constructed libraries were amplified using emPCR kits (Roche, IN, USA), and sequencing was then performed by 1/8 lane of the 454 GS FLX Titanium Sequencer at the National Instrumentation Center for Environmental Management (NICEM, Seoul National University). The sequence data generated in this study have been deposited at NCBI in the Short Read Archive database under the accession number SRA098802 (experiment accession number SRX338064). The data sets supporting the results of this article can be downloading at http://www.ncbi.nlm.nih.gov/sra/?term=SRA098802. The raw sequence reads generated were assembled by Newbler2.3 software (Roche) with 98% sequence similarity threshold.

### Functional annotation

To assess the quality of the *de novo* assembly, a similarity search against the NCBI nr protein database (ftp://ftp.ncbi.nlm.nih.gov/blast/db/FASTA/nr.gz), was conducted using the BLASTx algorithm with an E value threshold of 10^-5^. Further, all unigenes were searched against the NCBI non-redundant (nr) protein database (http://www.ncbi.nlm.nih.gov) for functional annotation using BLASTx with an e-value cutoff of 1e^-5^. The resulting BLAST hits were analyzed for the mapping step in order to retrieve Gene Ontology (GO) terms associated with the hits from the BLAST results. Subsequently, a GO annotation step to select GO terms from the GO pool obtained from the mapping step was performed by the Blast2GO program [78].

### Marker development

#### Selection of EST sequences containing SSRs and primer design

The MIcroSAtellite identification tool (MISA) at http://pgrc.ipk-gatersleben.de/misa/misa.html was used for detection of simple sequence repeats (SSRs). The criteria used for detection of EST sequences containing SSRs was a minimum of six repeats for dinucleotide motifs, five repeats for trinucleotide motifs and four repeats for tetra-, penta- and hexa- nucleotide motifs. EST sequences containing SSRs (SSR-ESTs) of cabbage C1234 was BLAST searched against EST sequences of cabbage C1184 (susceptible to black rot disease) using our local database (http://im-crop.snu.ac.kr/). After comparison, only C1234-unique SSR-ESTs, found in C1234 but not in C1184, were used for primer design. Primer pairs were designed for all selected SSR-ESTs from the flanking sequences of SSR motif using the Primer3 program (http://primer3.wi.mit.edu/). The parameters used for primer design were: 55–65°C melting temperature (Tm) with an optimum Tm of 60°C, primer length ranging from 18–24 nt with an optimum size of 20, GC content between 40% and 70% with an optimum set to 50% and product size estimated from 100 to 350 bp. The newly developed EST-SSR markers were designated with the BoESSR (*Brassica oleracea* EST-based SSR) prefix (e.g., BoESSR001, BoESSR002, BoESSR003, etc.).

#### SNP discovery and primer design

SNP identification was accomplished by CLC mapping of two cabbage parental lines, C1184 and C1234. Raw reads of C1234 were mapped onto C1184 contigs that were used as reference. In order to improve the accuracy of SNPs, the detected SNPs were then filtered based on the criteria of a minimum 70% of read depth. The selected SNPs were used to develop dCAPS markers using the dCAPS Finder 2.0 program (http://helix.wustl.edu/dcaps) for generation of nearly matched primers including SNP positions [79]. After designing mismatched primers for each SNP, the opposite primers were designed using the Primer3 program (http://primer3.wi.mit.edu/). All of the primers were synthesized by Macrogen (Seoul, Korea).

### Molecular marker analysis

A total of 3,570 markers were screened for detection of polymorphisms between the parental lines C1184 and C1234. Of these, 1,034 were EST-based markers comprising 937 EST-based SSR and 97 EST-based dCAPS markers that were developed in this study. Also included were 1,841 intron-based polymorphism (IBP) markers that were developed from *B. rapa* genome sequences [22]. Furthermore, 695 publically reported SSR markers were used to integrate the reference genetic map: 264 primers derived from the public domain [16,17], 94 primers from Wang et al. [14], 71 primers designed from publicly available *B. napus* genome survey sequences (GSSs) [20], 45 primers isolated from *B. napus*[23,29], 41 primers from Agriculture and Agri-Food Canada [20], 35 primers obtained from Burgess et al. [24], 27 primers designed from a microsatellite-enriched genomic library of *B. rapa*[25], 24 BAC-derived SSR primers from Kim et al. [21], 21 primers developed from *B. rapa* by Choi et al. [30], 18 primers from EST sequences of *B. napus*[31], 14 primers from Long et al. [18], 12 primers developed by Iniguez-Luy et al. [32], 11 primers originally isolated from *B. napus*[33,34], 9 database sequence-derived primers from Louarn et al. [26], 4 primers isolated from an *A. thaliana* library [35], and one primer each from Lagercrantz et al. [36], Sebastian et al. [37], Smith and King [15], HRI (unpublished data) and Saito et al. [38].

#### SSR and IBP analysis

PCR amplifications were performed in a total volume of 10 μL containing 10 ng DNA template, 1X PCR reaction buffer (Inclone Biotech), 0.2 mM each dNTP (Inclone Biotech), 0.2 μM each primer and 1 unit *Taq* DNA polymerase (Inclone Biotech). The PCR profile was as follows: initial denaturation at 94°C for 4 min, and then 35 cycles of 30 s denaturation at 94°C, 30 s annealing at 55°C - 60°C, 30 s extension at 72°C, and 10 min at 72°C for final extension. The PCR-amplified products were separated by 6% non-denaturing polyacrylamide gel electrophoresis using 1X TBE buffer. The gels were stained with ethidium bromide for 20 min and DNA bands were visualized under UV light using a gel documentation system. The PCR products of some markers were genotyped using Fragment Analyzer, an automated capillary electrophoresis system (Advanced Analytical Technologies Inc., USA), in order to obtain clear separation. The genotyping results were analyzed using *PROSize*^TM^ 2.0 analytical software, which can easily screen electropherograms or digital images using the flagging feature (Advanced Analytical Technologies Inc., USA).

#### dCAPS analysis

PCR reactions were performed in a total volume of 25 μL containing 20 ng DNA template, 0.2 μM each primer set, 1 × PCR buffer, 0.2 mM each dNTP, 1 unit *Taq* DNA polymerase (VIVAGEN, Korea). Amplification was conducted as described above. The amplified PCR products were digested with appropriate restriction enzymes (3 units) in the presence of the appropriate 1 × buffer, 1 × BSA (if necessary), and distilled water, with incubation at 37°C for more than 3 hours. The products were analyzed using 9% non-denaturing polyacrylamide gel electrophoresis and visualized on a UV trans-illuminator after ethidium bromide staining.

### Linkage analysis and map construction

Reproducible polymorphic markers were scored in the F_2_ population. Linkage analysis and map construction were performed using JoinMap version 3.0 [80]. Linked loci were grouped in the LOD (logarithm of odds) with minimum scores of 2.0, and linkage groups were assigned as C01 to C09, corresponding to the formerly reported map of this species. Locus order within the LOD grouping was generated for each linkage group using a recombination frequency below 0.4 and an LOD score above 0.5 for all marker pairs within each linkage group. The Kosambi function was used to convert recombinant values to genetic distances between the markers [81]. Seventy-five SSR markers derived from the reference map were used as anchor markers in order to assign the newly designed markers in this study to specific linkage groups (LGs). The final genetic linkage map was drawn using MapChart [82].

## Competing interests

The authors declare that they have no competing interests.

## Authors’ contributions

NKI generated EST-based SSR markers, analyzed of previously reported SSR and IBP markers, constructed linkage map and drafted the manuscript. JL carried out transcriptome sequencing, generated EST-based dCAPS markers and edited the manuscript. MJ analyzed of transcriptomics data and edited the manuscript. SP participated in data analysis. KGA provided the plant materials used in this study. MJn and BSP provided IBP primers. TJY designed the study and edited the manuscript. All authors read and approved the final manuscript.

## Supplementary Material

Additional file 1 EST sequences related to NBS-LRR. Click here for file

Additional file 2 Description of EST-SSR markers polymorphic between cabbage C1184 and C1234. Click here for file

Additional file 3 Characterictics of EST-based dCAPS markers polymorphic between C1184 and C1234 parental lines. Click here for file
